# Design and Prototype of an Automated Column-Switching HPLC System for Radiometabolite Analysis

**DOI:** 10.3390/ph9030051

**Published:** 2016-08-17

**Authors:** Neil Vasdev, Thomas Lee Collier

**Affiliations:** 1 Division of Nuclear Medicine and Molecular Imaging & Gordon Center for Medical Imaging, Department of Radiology, Massachusetts General Hospital, Harvard Medical School, Boston, MA 02114, USA; collierl@advion.com; 2 Advion Inc., Research and Development, Ithaca, NY 14850, USA

**Keywords:** positron emission tomography, radiometabolite, column-switching HPLC, plasma

## Abstract

Column-switching high performance liquid chromatography (HPLC) is extensively used for the critical analysis of radiolabeled ligands and their metabolites in plasma. However, the lack of streamlined apparatus and consequently varying protocols remain as a challenge among positron emission tomography laboratories. We report here the prototype apparatus and implementation of a fully automated and simplified column-switching procedure to allow for the easy and automated determination of radioligands and their metabolites in up to 5 mL of plasma. The system has been used with conventional UV and coincidence radiation detectors, as well as with a single quadrupole mass spectrometer.

## 1. Introduction

Positron emission tomography (PET) radioligands designed to image the central nervous system (CNS) are most commonly labeled with carbon-11 or fluorine-18 which have short half-lives of 20.4 and 109.7 min, respectively. Obtaining quantitative measures with such radioligands in plasma samples, containing low amounts of radioactivity is critical in clinical research and drug development studies to accurately estimate concentrations of neuroreceptors, transporters or enzymes in vivo. Among the many challenges of developing and routinely utilizing CNS radiotracers for in vivo studies is the accurate assessment of radiotracer metabolism. Unfortunately, it is very rare for neuro-PET radiotracers to resist metabolism to “troublesome” radiometabolites during the course of a PET scan [1,2]. It is well know that intravenously administered radiotracers are immediately exposed to biological processes that start metabolizing the radiotracers such that the fraction of radioactivity present in blood as unchanged radiotracer quickly decreases. Radiometabolites are usually less lipophilic than the parent radiotracer and normally cannot cross the blood brain barrier. However, some radiometabolites can either cross the blood brain barrier or generate in the brain. PET and single-photon emission computed tomography cameras have no means to discriminate between the chemical identity of detected radioactivity. An ideal radiotracer would not undergo any metabolism, however some metabolites actually bind more efficiently to the target receptor or another target. It is imperative that radiometabolites following administration of a radiotracer in vivo are assessed in order to ensure that appropriate biomathematical analyses and kinetics of ligand binding to the respective target can be determined.

Several methods have been employed to measure radiometabolites in blood, plasma or brain extracts. For ^11^C- and ^18^F-labeled radiotracers designed for imaging the CNS, radiochemical analysis of metabolites from plasma is most commonly conducted using either high performance liquid chromatography (HPLC) or solid phase extraction. Hilton and coworkers developed a manual column-switching HPLC system for PET radiotracer analysis where plasma is directly loaded onto a capture column from which the analyte is subsequently eluted onto a reverse-phase analytical column [3]. This system has been extensively adopted by the neuro-PET community as the major advantages include decreasing the time-consuming and labor intensive sample preparation and manual manipulation of the radioactive samples. This system also completely accounts for all radioactivity loaded in the plasma sample. Wilson and coworkers modified the original design [3] and applied it to analyze plasma from several ^11^C- and ^18^F-labeled radiotracers as well as extracts from brain homogenates. The lack of streamlined apparatus and consequently varying protocols remain as a challenge among PET laboratories and the main objective of the present work was to improve the reliability and reproducibility of such column-switching HPLC systems. Herein we present an automated prototype column-switching HPLC apparatus that can be easily constructed for improvement of inter-laboratory processing of radiometabolite data.

## 2. Results and Discussion

Plasma was obtained by centrifugation of whole blood or using a Vivid™ Plasma Separation Membrane (Pall Corp., Westborough, MA, USA) [5]. The plasma separation membrane is currently being investigated and offers an advance towards the complete automation of this process as the manual centrifugation step and manipulation of the radioactive sample can be avoided.

**Figure 1:**
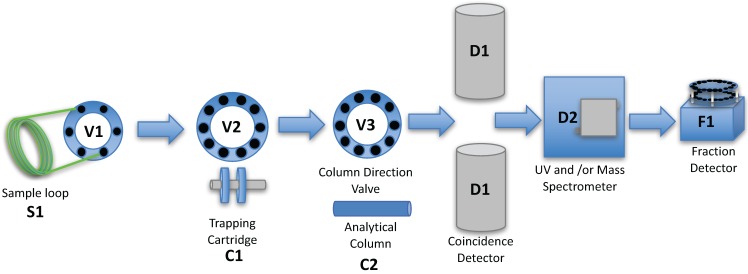
Flow chart of the processing of a metabolite sample. The plasma sample loaded on to the Sample loop S1 is transferred to the trapping cartridge C1. The materials retained on the trapping cartridge are then separated on the Analytical column C2. All materials from the trapping cartridge and the analytical column are passed through the coincidence detector D1 and UV and/or Mass Spectrometer D2 and collected in the fraction collector F1 for analysis in a well counter.

Modified literature procedures [3,4] were used to design the automated column-switching HPLC apparatus (Figure 1). The system consists of two HPLC pumps (Waters 515, Waters Corp., Milford, MA, USA), a HPLC injector with a 1–5 mL sample loop, and two 10 port 2-position valves. One of the valves is used to concentrate the metabolites and separate the proteins onto the capture column containing a suitable media (typically Waters™ Oasis HLB). Once switched, the material trapped on the cartridge is backflushed, and directly applied to an analytical column connected in series (typically C-18), to separate the metabolites, which are detected via a coincidence gamma detector (Bioscan, Washington, D.C., USA).

Because over-pressurization, as measured by the HPLC pump transducer, of either the capture column and/or the analytical HPLC column are common problems that lead to incomplete radiometabolite analyses, a second valve is used to rapidly reverse the flow paths, thereby “back-flushing” any analytes trapped on the analytical column. In a similar manner, the components may be analyzed via a coincidence gamma detector and/or mass spectrometer. Over-pressurization of the analytical column is internally monitored, either by the pumps internal pressure gauge, or via an external pressure transducer. When system detects that the HPLC pump pressure exceeds a preset limit (typically 3500 psi; monitored through LabView™ software (National Instruments Corporation, Austin, TX, USA)), it will automatically back-flush the analytical column.

**Figure 2:**
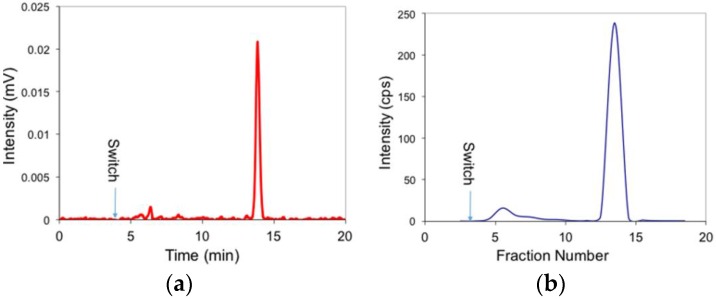
Comparison of high performance liquid chromatography (HPLC) chromatogram and plasma fractions counted in a gamma counter for a [^18^F] flurpiridaz sample. (**a**) Raw chromatogram from inline coincidence detector (60% CH_3_CN in phosphate buffered saline eluted through a C-18 column (Waters Xbridge BEH, 130Å; 100 × 4.6 mm, 3.5 μm) at 1 mL/min; (**b**) 2 mL fractions from the HPLC analysis were counted on the gamma counter, then plotted as smoothed line for comparison.

The limit of detection of the coincidence detector (SNR ≥ 5) was assessed using serial dilutions of a carbon-11 solution and was determined to be ~11 nCi injected. A blood sample obtained at 25 min post injection in a ^18^F-radiotracer solution was centrifuged and then 5 mL of the plasma layer was injected onto the system. The measured chromatogram and fractions are shown in Figure 2.

As centrifugation of blood requires manual manipulations and therefore poses an additional source of error in these experiments, we are currently developing a simple sample preparation methodology that takes advantage of a plasma separation membrane. This membrane system aims to eliminate the need for centrifugation of whole blood and would enable complete automation of the column-switching HPLC process, and is the focus of our future work.

This work has demonstrated the utility of the radiometabolite analysis system as follows: (a) A prototype apparatus for automated column-switching HPLC analysis of metabolites has been designed; (b) The present system minimizes operator errors and does not require manual column-switching; (c) Over-pressurization is internally monitored and the system automatically back-flushes the analytical column; (d) The use of the removable trapping cartridge allows for rapid change of cartridges when over pressurization is detected.

## 3. Experimental Section

The system followed the basic apparatus and design outlined in the papers of Hilton [3] and Wilson [4], with modifications to allow for the automation of the process and to eliminate the need to start and stop pumps and the need to switch two 6-port valves [6]. The HPLC system consists of two Waters 515 pumps, one delivering 1% acetonitrile in water to the 1–5 mL loop of a 7725i injector (IDEX Health and Science, Middleborough, MA, USA) at 1 mL/min and the other delivering the analytical mobile phase required for the separation of the compound of interest at the required flow rate and using the required mobile phase for the separation. The configuration of columns and valves, both automated and manual, used for the automated column-switch HPLC is shown in Figure 1. A 1–5 mL sample is loaded into the injector loop S1 of a 7725i 2-position sample injection valves (V1, Rheodyne LLC, Rohnert Park, CA, USA). At the time of injection, a VXV PEEK Analytical High Pressure 2-position, 10 Port Switching Valve (V2, Analytical Sales and Services, Pompton Plains, NJ, USA) directs the plasma sample, displaced from the injector by 1% acetonitrile, to the capture column C1, while at the same time the same ten-port valve V1, directs the C1 effluent to the radioactivity detector D1 (Figure 1). The detector contains two ~0.25 mL loops positioned between two opposing bismuth germanium oxide (BGO) detectors (Bioscan Inc., Washington, D.C., USA). One loop is used to detect the material not trapped by the trapping cartridge C1, the second loop is attached to the output of the analytical HPLC column. During the first 4 min, the detector responds to radioactive species in the plasma that are not trapped by the sorbent of the capture column, C1. Four minutes after the injection, V2 is switched to allow the analytical mobile phase to back-flush the contents of C1 onto the analytical column C2 and the effluent from the analytical column is allowed to flow past the radiation detector D1 and UV and/or Mass spectrometer D2. At the end of the HPLC elution time from the analytical column, V2 is returned to its original position, which allows the capture column C1 to be washed with 1% acetonitrile in preparation for next plasma sample. If the pressure of the analytical column exceeds a pressure limit (typically 3500 psi), the analytical column can be automatically reversed using a VXV PEEK Analytical High Pressure 2-position, 10 Port Switching Valve (V3, Analytical Sales and Services, Pompton Plains, NJ, USA). The capture column (C1, Figure 1) measuring 4 × 20 mm (VICI part number SFEC42) is packed dry with C-18, or HLB Sep-pak sorbent (Waters Corp., Milford, MA, USA) and is packed by vibration to remove voids and is contained in a guard column housing (Part No.SFECH412, VICI) with 2µ titanium frits. The output of the HPLC column can be directed to a Mass Spectrometer D2 either directly or through a split with the remaining material going to a fraction collector F1 (Spectra/Chrom™ CF-1 Fraction Collector (SpectrumLabs, Rancho Dominguez, CA, USA). The samples from the fraction collector are then assayed for radioactive content on a Perkin Elmer/Wallac Wizard™ 1470 Automatic Gamma Counter (Perkin Elmer, Waltham, MA, USA). The signal from the coincidence detector is collected by a PowerChrom 280 system (which includes software and data acquisition software, eDAQ Pty Ltd, Denistone East, NSW, Australia) The same software is used to integrate the peak areas and to control the position of the valves.

**Figure 3:**
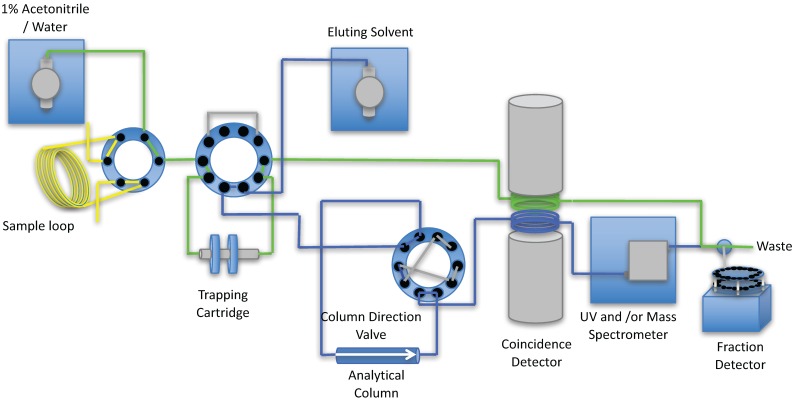
Equilibration of the trapping cartridge and analytical column.

**Figure 4:**
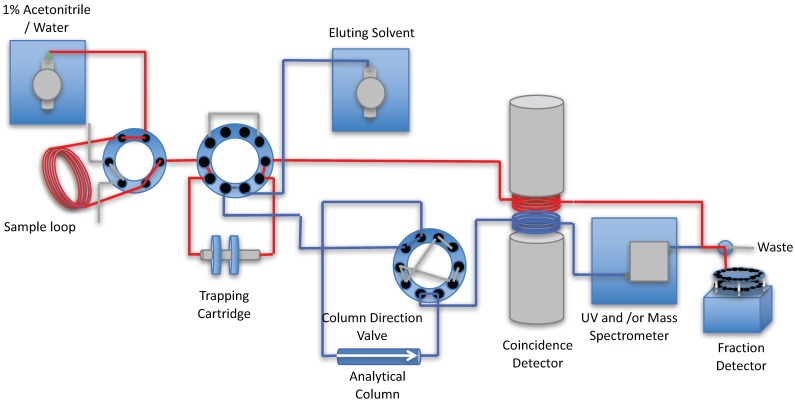
The loading of the plasma from the sample loop to the trapping cartridge while the analytical column continues to be equilibrated with the eluting solvent.

**Figure 5:**
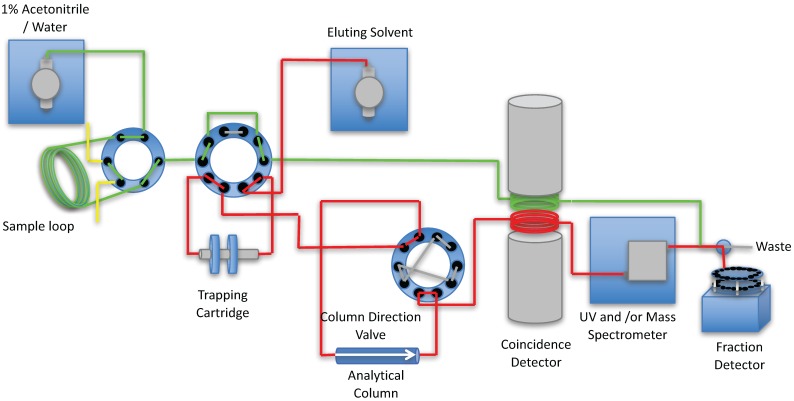
The elution of the plasma sample from the trapping cartridge to the analytical column which is analyzed by the coincidence detector, UV detector and/or mass spectrometer.

**Figure 6:**
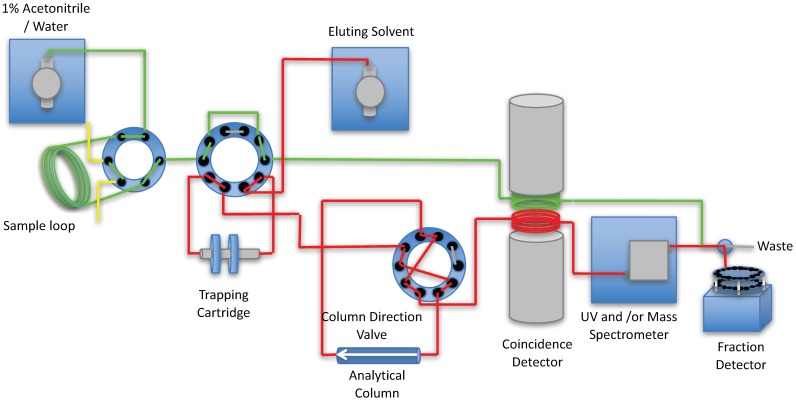
If the pressure of the analytical column exceeds the set pressure limit, the 10-port column direction valve V3 is switched, the analytical column flow is reversed to remove materials obstructing the flow of solvent. Once the pressure drops the valve is switched and the flow returned to normal flow, as per Step 3.

A schematic of the processing of a typical metabolite sample is shown in Figure 3, Figure 4, Figure 5 and Figure 6.

Step 1. The plasma is injected onto the injection loop (Yellow line), either manually or by autosampler, while the trapping cartridge (Green line) is equilibrated with the wash solvent (typically 1% acetonitrile/water) and the analytical column (Blue line) is equilibrated with the elution solvent. All solvent is passed to waste and bypasses the fraction collector.

Step 2. The 6-port valve with the sample loop containing the plasma sample is transferred to the trapping cartridge (Red line). The analytical column (Blue line) continues to be equilibrated with the elution solvent. All solvent is passed to fraction collector so any material that is not trapped on the cartridge is collected in the fraction collector.

Step 3. The 10-port valve with the analytical column is switched and the plasma sample is eluted from the trapping cartridge to the analytical column (Red line). The sample loop (Green line) continues to be washed with the 1% acetonitrile/water solution to clean prior to the next sample being loaded. All solvent is passed to fraction collector so all material that is not detected by the coincidence detector can be counted using a well counter.

Step 4. If the back-pressure on the analytical column exceeds a set limit, the 10-port valve V3 changes position so that the column flow is reversed. This back-flushing cleans the column and after a set time the forward flow is restored.

## Abbreviations

The following abbreviations are used in this manuscript:

CNS: Central nervous system
PET: Positron emission tomography
HPLC: High performance liquid chromatography
UV: Ultraviolet
